# EAB-BES: A Global Optimization Approach for Efficient UAV Path Planning in High-Density Urban Environments

**DOI:** 10.3390/biomimetics10080499

**Published:** 2025-07-31

**Authors:** Yunhui Zhang, Wenhong Xiao, Shihong Yin

**Affiliations:** 1 School of Internet, Jiaxing Vocational and Technical College, Jiaxing 314036, China; yunhuizhang@jxvtc.edu.cn; 2 Jiaxing Key Laboratory of Industrial Internet Security, Jiaxing Vocational and Technical College, Jiaxing 314036, China; 3 School of Automation, Nanjing University of Science and Technology, Nanjing 210094, China

**Keywords:** unmanned aerial vehicle, path planning, bald eagle search algorithm, multi-strategy enhanced, block-based elite-guided differential mutation, metaheuristic

## Abstract

This paper presents a multi-strategy enhanced bald eagle search algorithm (EAB-BES) for 3D UAV path planning in urban environments. EAB-BES addresses key limitations of the traditional bald eagle search (BES) algorithm, including slow convergence, susceptibility to local optima, and poor adaptability in complex urban scenarios. The algorithm enhances solution space exploration through elite opposition-based learning, balances global search and local exploitation via an adaptive weight mechanism, and refines local search directions using block-based elite-guided differential mutation. These innovations significantly improve BES’s convergence speed, path accuracy, and adaptability to urban constraints. To validate its effectiveness, six high-density urban environments with varied obstacles were used for comparative experiments against nine advanced algorithms. The results demonstrate that EAB-BES achieves the fastest convergence speed and lowest stable fitness values and generates the shortest, smoothest collision-free 3D paths. Statistical tests and box plot analysis further confirm its superior performance in multiple performance metrics. EAB-BES has greater competitiveness compared with the comparative algorithms and can provide an efficient, reliable and robust solution for UAV autonomous navigation in complex urban environments.

## 1. Introduction

In recent years, unmanned aerial vehicles (UAVs) have proliferated in various fields, including aerial photography [1], surveillance [2,3], delivery services [4] and disaster relief operations [5,6]. Their capability to operate in three-dimensional (3D) spaces provides unique advantages in scenarios where traditional ground-based or two-dimensional (2D) systems are insufficient.

However, UAV navigation in complex environments faces significant challenges due to obstacles such as mountains, buildings, and power lines [7,8], posing risks and restricting UAV operations. UAV path planning aims to determine optimal or near-optimal trajectories that avoid obstacles and satisfy constraints. The complexity of path planning significantly increases in 3D environments compared to 2D scenarios [9].

Many existing UAV path planning algorithms, such as A* [10], rapidly exploring random trees (RRT) [11,12], and probabilistic roadmaps (PRM) [13], heavily rely on pre-generated global environment maps [14]. This dependency makes real-time path planning difficult, especially in dynamic and large-scale environments [15,16]. Furthermore, the computational complexity increases exponentially with search space expansion, limiting real-time applicability [17].

Nature-inspired swarm intelligence algorithms offer promising alternatives due to their adaptability, distribution, and self-organization characteristics [18,19,20,21]. Among these, the Bald Eagle Search (BES) algorithm [22], inspired by bald eagle foraging behavior, has gained attention for its simplicity and powerful global search capability. BES balances exploration and exploitation through selection, search, and swooping stages. Despite its strengths, BES still suffers from slow convergence and susceptibility to local optima, limiting its effectiveness in complex urban environments. Nature-inspired swarm intelligence algorithms offer promising alternatives due to their adaptability, distribution, and self-organization characteristics [18,19,20,21]. Among these, the Bald Eagle Search (BES) algorithm [22], inspired by bald eagle foraging behavior, has gained attention for its simplicity and powerful global search capability. BES balances exploration and exploitation through selection, search, and swooping stages. Despite its strengths, BES still suffers from slow convergence and susceptibility to local optima, limiting its effectiveness in complex urban environments. Inspired by biological behaviors, BES belongs to the family of biomimetic algorithms that model animal foraging and survival strategies to solve complex computational problems. This study builds on this biomimetic foundation by enhancing BES with biologically plausible strategies that mimic elite guidance, adaptive response, and localized mutation, thereby improving its performance in challenging UAV path planning scenarios.

In this work, we propose an enhanced version of BES named EAB-BES, which incorporates three strategies: elite opposition-based learning, adaptive weight mechanism, and block-based elite-guided differential mutation. And apply the EAB-BES algorithm to the three-dimensional path planning problem of unmanned aerial vehicles in urban environments. The innovation of this work lies in the strategic integration and architectural synergy of these mechanisms within the BES algorithm framework to overcome its specific limitations. The three-stage structure of BES itself is more suitable for urban hierarchical path modeling. This significantly improves the performance of complex 3D UAV path planning. By introducing these strategies, EAB-BES significantly improves convergence speed, path accuracy, and obstacle avoidance capabilities in urban settings. Traditional BES is prone to getting stuck in local solutions during the swooping phase, especially in the synthesis strategy, where the block-based elite-guided differential mutation strategy generates directional perturbation vectors through subgroup competition, making it easy to escape from local optima. The key contributions of this paper are as follows:

- Developed a specialized 3D UAV path planning model addressing collision avoidance and navigation efficiency among mixed-height urban obstacles.
- Proposed the EAB-BES algorithm, integrating elite opposition-based learning, adaptive weighting, and block-based elite-guided differential mutation to enhance BES’s convergence speed, accuracy, and adaptability.
- Introduced a novel block-based elite-guided differential mutation strategy for dynamically refining population subgroups, improving local optima escape ability and path robustness.
- Validated the performance of EAB-BES through comparative experiments with 9 state-of-the-art algorithms in six high-density urban scenarios.

The rest of the paper is organized as follows: Section 2 introduces the related work. Section 3 describes preliminary concepts, including the UAV path planning problem and the basic BES algorithm. Section 4 details the proposed EAB-BES algorithm. Section 5 presents experimental results and discussions. Section 6 provides conclusions and future research directions.

## 2. Related Work

Research in UAV path planning has gained considerable attention due to increasing applications and complexity. Zeng et al. [23] proposed an energy-efficient communication strategy between UAVs and ground terminals. Kim et al. [24] developed a real-time UAV path planning algorithm using compensated Voronoi diagrams suitable for challenging environments like disaster areas and battlefields. Ergezer et al. [25] introduced a novel evolutionary operator for single UAV path planning, and Jayaweera et al. [26] adopted an artificial potential field (APF) method ensuring stable tracking of moving ground targets under windy conditions. Ikram et al. [27] proposed sequential block path planning (SBPP), while Fahmani et al. [28] developed a trajectory optimization method combined with UAV-based transmission line detection. Pamarthi et al. [29] designed a UAV motion planning framework for dynamic environments. Chan et al. [30] proposed an energy-efficient path planning model for UAVs navigating urban terrain influenced by wind.

Recently, numerous swarm intelligence algorithms have been applied to UAV path planning. For example, Roberge et al. [31] used genetic algorithm (GA) and particle swarm optimization (PSO) to generate feasible trajectories for fixed-wing UAVs in complex 3D scenarios. Liu et al. [32] presented a modified sparrow search algorithm (CASSA) for high-dimensional UAV route optimization. Zhao et al. [33] integrated the selfish herd optimizer (SHO) with PSO (SHOPSO) for UAV path planning, whereas Wang et al. [34] developed a modified mayfly algorithm (modMA) aimed at reducing path costs. Aslan et al. [35] designed a fast, real-time autonomous path planning approach for UAVs in large-scale environments. Yin et al. [36] proposed adaptive differential evolution with dynamic Thompson sampling (DE-DYTS) for collaborative multi-UAV path planning. Wang et al. [37] introduced an enhanced red-tailed hawk algorithm (IRTH) for practical UAV applications. Zhong et al. [38] extended reinforcement learning (RL) to cross-regional UAV path planning using improved Q-learning (QL). Zhang et al. [39] developed a multi-strategy ensemble wind-driven optimization (MEWDO) algorithm integrating cubic spline interpolation into robot path planning. Wang et al. [40] proposed an improved human evolutionary optimization algorithm (IHEOA) for UAV route optimization, and Yin et al. [41] developed a collaborative path planning strategy for unmanned surface vehicles in complex water environments with multi-stage constraints.

The Bald Eagle Search (BES) algorithm, inspired by the foraging behavior of bald eagles, has attracted significant attention due to its simplicity and strong global search capability. As a recently developed swarm intelligence algorithm [42], BES effectively balances exploration and exploitation through three stages: selection, search, and swooping. Its versatility has led to applications in diverse fields, such as engineering optimization [43,44], geometric approximation [45], biomedicine [46], mobile edge computing [47], energy storage [48], forest fire detection [49], face recognition [50], autonomous driving [51], path planning [52], water resource management [53], and IoT security [54]. However, BES still has limitations, such as slow convergence speed and susceptibility to local optima, which restrict its performance in complex urban UAV path planning tasks.

Despite the advantages of BES and other swarm intelligence algorithms, limitations remain regarding convergence speed, computational efficiency, and the ability to escape local optima. These challenges become particularly prominent in complex 3D urban environments. Therefore, further research into enhancing algorithmic performance through multi-strategy integration is essential to address these gaps.

## 3. Preliminary

### 3.1. Path Planning for UAV

UAVs are playing an increasingly crucial role in our daily lives. They have been widely used in mountainous environments, with preliminary research results achieved. However, in complex urban environments, the demands for urban logistics distribution, emergency rescue, and infrastructure inspection are growing rapidly. Given the numerous tall buildings, complex terrains, and abundant electromagnetic interference sources in cities, traditional path planning methods face difficulties. Thus, conducting UAV path planning becomes highly necessary. This paper focuses on researching UAV path planning in urban environments, aiming to enable UAVs to find safe and efficient flight paths in complex urban settings, enhance their application efficiency in urban scenarios, and ensure the smooth progress of urban-related operations.

In the context of urban-based UAV path planning, high-rise buildings and low-squat buildings are regarded as the primary obstacles to UAV flight. In a 3D urban environment, cylinders are employed to simulate high-rise buildings, while hemispheres are utilized to represent low-squat buildings.

**Figure 1:**
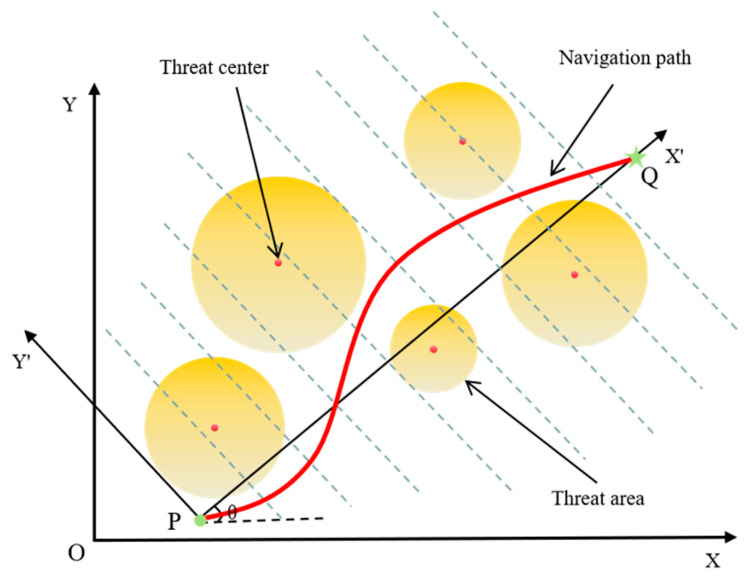
Top view of UAV path planning.

The top-view schematic diagram of the UAV’s navigation path is shown in Figure 1. P and Q are marked as the starting and goal positions, respectively. In complex urban environments, high-rise and low-rise buildings are the main obstacles, represented by circular projections with different radii and threat levels. If a part of the UAV’s flight path enters a circular threat area, the UAV will be threatened. When the flight path does not pass through the circular projection, the path is safe. Therefore, the goal of this UAV is to obtain the optimal path from P to Q while minimizing costs as much as possible.

Our fitness function aims to comprehensively consider factors such as path length, flight altitude, and building threats to evaluate the quality of UAV paths. The fitness function in this article mainly consists of three parts: path length cost, flight height cost, and threat cost.

(1) Path Length Cost ($F_{lengthCost}$)

Suppose the coordinate sequence of the interpolation points is $\left(x_{1}(i),y_{1}(i),z_{1}(i)\right)$, where i=1,2,…,n, and n is the number of interpolation points. The path length cost is obtained by calculating the sum of the Euclidean distances between adjacent interpolation points, and the formula is

(1) $$F_{lenghtCost}=\sum_{i=1}^{n-1}\sqrt{{\left(x_{1}(i+1)-x_{1}(i)\right)}^{2}+{\left(y_{1}(i+1)-y_{1}(i)\right)}^{2}+{\left(z_{1}(i+1)-z_{1}(i)\right)}^{2}}.$$

(2) Flight Height Cost ($F_{heightCost}$)

The flight height cost consists of two parts:

Height deviation cost of intermediate navigation nodes ($F_{heightCost_{1}}$): Suppose the coordinate sequence of the navigation nodes is $\left(X_{s}(i),Y_{s}(i),Z_{s}(i)\right)$, where i=1,2,…,m, and m is the number of navigation nodes. The optimal flight height $BestFliH\left(i\right)$ is obtained based on terrain interpolation, and the calculation formula is

(2) $$BestFliH(i)=\mathrm{interp}2(X,Y,Z,X_{s}(i),Y_{s}(i))+Optimal\_h,$$

where Optimal_h=500 is the offset of the optimal flight height relative to the ground. Then the height deviation cost of intermediate navigation nodes is

(3) $$F_{heightCost_{1}}=\sum_{i=2}^{m-1}\frac{\left|Z_{s}(i)-BestFliH(i)\right|}{BestFliH(i)}.$$

Flight height constraint penalty cost ($F_{heightCost_{2}}$): The minimum flight height $MinFliH\left(i\right)$ is obtained based on terrain interpolation, and the calculation formula is

(4) $$MinFliH(i)=\mathrm{interp}2(X,Y,Z,x_{1}(i),y_{1}(i))+Min\_h,$$

where Min_h=10 is the offset of the minimum flight height relative to the ground. When $z_{1}(i)<MinFliH\left(i\right)$ or $\mathrm{isnan}(MinFliH\left(i\right))$, a penalty value $Penaltyf=1\times 10^{6}$ is applied; otherwise, it is 0. Then the flight height constraint penalty cost is

(5) $$F_{heightCost_{2}}=\left\{\begin{array}{ll} Penaltyf, & \;\mathrm{if}\;z_{1}(i)<MinFliH(i)\;\mathrm{or}\;\mathrm{isnan}(MinFliH(i))\\ 0, & \mathrm{otherwise} \end{array}\right..$$

Therefore, the total flight height cost is

(6) $$F_{heightCost}=F_{height\mathrm{Cos}t_{1}}+F_{height\mathrm{Cos}t_{2}}.$$

(3) Threat Cost ($F_{threatCost}$)

Let $Threat_{A}(i)$ represent the threat value of the *i*-th interpolation point from high-rise buildings, and $Threat_{R}(i)$ represent the threat value of the *i*-th interpolation point from low-rise buildings. $Penaltyt=1\times 10^{3}$ is the threat penalty factor. Then the calculation formula for the threat cost is

(7) $$F_{threatCost}=Penaltyt\sum_{i=1}^{n}(Threat_{A}(i)+Threat_{R}(i)).$$

Threat value from high-rise buildings ($Threat_{A}(i)$): Suppose the number of high-rise buildings is p. For the *j*-th high-rise building, define its base center coordinates as $\left(x_{j},y_{j},z_{j}\right)$, its base radius as $r_{j}$ and its height as $h_{j}$. The threat value for the *i*-th path point is computed if the conditions below are satisfied.

Horizontal distance constraint: The horizontal projection distance from the path point to the building base must be less than the radius $r_{j}$:

(8) $$\sqrt{{\left(x_{1}(i)-x_{j}\right)}^{2}+{\left(y_{1}(i)-y_{j}\right)}^{2}}\leq r_{j}.$$

Vertical height constraint: The vertical coordinate of the path point must lie within the building’s height range:

(9) $$z_{1}(i)\in [z_{j},z_{j}+h_{j}].$$

The threat value is formulated as follows:

(10) $$Threat_{A}(i)=\left\{\begin{array}{ll} \sum_{j=1}^{p}\max\left\{r_{j}-\sqrt{{\left(x_{1}(i)-x_{j}\right)}^{2}+{\left(y_{1}(i)-y_{j}\right)}^{2}},0\right\}, & \mathrm{if}\;z_{1}(i)\in [z_{j},z_{j}+h_{j}]\\ 0, & otherwise \end{array}\right..$$

Threat value from low-rise buildings ($Threat_{R}(i)$): Suppose the number of low-rise buildings is l. For the *j*-th high-rise building, define the center coordinates of the hemisphere as $\left(x_{j},y_{j},z_{j}\right)$, with radius $r_{j}$. The threat value for the *i*-th path point is computed if the conditions below are satisfied.

Three-dimensional distance constraint: The Euclidean distance from the path point to the hemisphere center must be less than the radius $r_{j}$:

(11) $$\sqrt{{\left(x_{1}(i)-x_{j}\right)}^{2}+{\left(y_{1}(i)-y_{j}\right)}^{2}+{\left(z_{1}(i)-z_{j}\right)}^{2}}\leq r_{j}.$$

Vertical height constraint: The path point must lie within the upper half-space covered by the hemisphere:

(12) $$z_{1}(i)\geq z_{j}.$$

The threat value is formulated as follows:

(13) $$Threat_{R}(i)=\sum_{j=1}^{l}\left\{\begin{array}{ll} \max\left\{r_{j}-\sqrt{{\left(x_{1}(i)-x_{j}\right)}^{2}+{\left(y_{1}(i)-y_{j}\right)}^{2}+{\left(z_{1}(i)-z_{j}\right)}^{2}},0\right\}, & \mathrm{if}\;z_{1}(i)\geq z_{j}\\ 0, & \mathrm{otherwise} \end{array}\right..$$

In order to balance the above three parts, allocation weights $\lambda_{1},\;\lambda_{2},\;\lambda_{3}$ are set, each being 1/3. The fitness function is

(14) $$Fitness=\lambda_{1}F_{lengthCost}+\lambda_{2}F_{heightCost}+\lambda_{3}F_{threatCost}.$$

### 3.2. Bald Eagle Search Algorithm

During the selection phase, the bald eagle accurately locates and selects the most advantageous position within the specified search space. This choice is based on the amount of food available in each region. The mathematical expression is

(15) $$X_{i,new}=X_{\mathrm{best}}+\varepsilon \cdot r(X_{\mathrm{mean}}-X_{i}),$$

where $X_{i,new}$ refers to the updated position of the *i*-th bald eagle. $X_{\mathrm{best}}$ indicates the current optimal position of the bald eagle. The position change parameter ε has a value in the range (1.5, 2), and r is a random number in the open interval (0, 1). Meanwhile, $X_{\mathrm{mean}}$ represents the average position of the bald eagle, and $X_{i}$ denotes the *i*-th position of a bald eagle.

After the selection phase is completed, BES enters the search phase. The algorithm systematically searches for prey within a previously determined area by mimicking the hunting actions of bald eagles. The eagle moves in a circle and gradually expands its search range in a spiral pattern. The mathematical description is

(16) $$X_{i,new}=X_{i}+y(i)\cdot (X_{i}-X_{i+1})+x(i)\cdot (X_{i}-X_{\mathrm{mean}})$$

(17) $$\left\{\begin{array}{c} x(i)=\frac{xr(i)}{\max(\left|xr\right|)}\\ y(i)=\frac{yr(i)}{\max(\left|yr\right|)} \end{array}\right.$$

(18) $$\left\{\begin{array}{c} xr(i)=r(i)\cdot \sin\left(\theta (i)\right)\\ yr(i)=r(i)\cdot \cos\left(\theta (i)\right) \end{array}\right.$$

(19) $$\left\{\begin{array}{l} \theta (i)=a\cdot \pi \cdot rand\\ r(i)=\theta (i)+R\cdot rand \end{array}\right..$$

In polar space, x(i) and y(i) represent the position of the *i*-th bald eagle. Both values are restricted to the interval between −1 and 1. $X_{i+1}$ is the subsequent updated position of the *i*-th bald eagle. θ(i) and r(i) are the polar angle and polar diameter, respectively. The spiral parameters a and R fall within the intervals (5, 10) and (0.5, 2), respectively. Additionally, rand is a random number with a value ranging from 0 to 1.

During the swooping phase, the bald eagle will dive to catch prey locked in search space. Equation (20) describes this behavior.

(20) $$X_{i,new}=rand\cdot X_{\mathrm{best}}+x1(i)\cdot (X_{i}-c_{1}\cdot X_{\mathrm{mean}})+y1(i)\cdot (X_{i}-c_{2}\cdot X_{\mathrm{best}})$$

(21) $$\left\{\begin{array}{c} x_{1}(i)=\frac{xr(i)}{\max(\left|xr\right|)}\\ y_{1}(i)=\frac{yr(i)}{\max(\left|yr\right|)} \end{array}\right.$$

(22) $$\left\{\begin{array}{c} xr(i)=r(i)\cdot \sinh\left(\theta (i)\right)\\ yr(i)=r(i)\cdot \cosh\left(\theta (i)\right) \end{array}\right.$$

(23) $$\left\{\begin{array}{l} \theta (i)=a\cdot \pi \cdot rand\\ r(i)=\theta (i) \end{array}\right..$$

The BES algorithm incorporates enhancement coefficients $c_{1}$ and $c_{2}$, with each coefficient having a value within the range of 1 to 2. To gain a more thorough understanding of the BES algorithm, the pseudocode is presented in Algorithm 1.
**Algorithm 1:** The pseudo-code of BES**Input:** *N* (population size), *D* (dimension), *Maxiter* (Maximum number of iterations), *up*, *lb* (upper and lower bounds) 1. Randomly generated initial point $X_{i}$ 2. Evaluate the fitness values 3. **while** (*t* < *Max_iter*) 4.     **for** *i* = 1 to *N*        **Select stage** 5.       Update individual position $X_{i}$ by Equation (15)        **Search stage** 6.       Update individual position $X_{i}$ by Equation (16)        **Swooping stage** 7.       Update individual position $X_{i}$ by Equation (20) 8.     **end for** 9. **end while** 10. **Output:** the optimal solution

## 4. Proposed EAB-BES Algorithm

This paper proposes a multi-strategy enhanced bald eagle search algorithm (EAB-BES) that integrates three critical innovations to address optimization limitations. First, an elite opposition-based learning mechanism is used for population initialization, employing bidirectional search to enhance diversity and accelerate convergence. Second, an adaptive weight factor is introduced into the iterative framework, dynamically balancing global exploration and local exploitation. Third, a block-based elite-guided differential mutation strategy partitions the population into competitive subpopulations, generating directional perturbation vectors. These vectors are modulated by a nonlinear convergence factor to balance exploration–exploitation trade-offs. By incorporating elite guidance into mutation, the strategy preserves diversity while refining the best solution, helping to avoid local optima and improving solution accuracy during fine-tuning.

### 4.1. Elite Opposition-Based Learning Strategy

Before the iterative optimization begins, the bald eagle population’s positions are randomly initialized. However, this randomness may expand the feasible solution range and increase search time, as the optimal solution’s location remains unknown. Population initialization significantly impacts search performance. To address this, an elite opposition-based learning (EOBL) strategy [55] is introduced. The inverse solutions (candidate solutions) are generated within the elite group’s range, and the population is updated by selecting the better solutions. This approach enhances search space exploration, improves diversity, and accelerates convergence.

**Figure 2:**
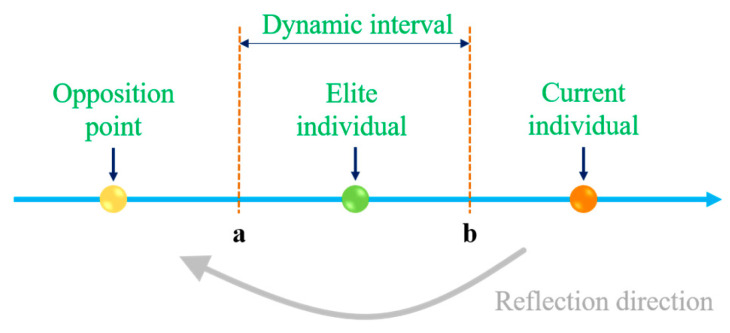
Elite opposition-based learning.

EOBL introduces the dynamic information of elite individuals on the basis of basic opposition-based learning (OBL) and expands the generation interval of opposition solutions from a fixed range to a dynamic interval based on the elite group. The specific steps are as follows:

(1) Selection of the elite group: Select the top 10% of individuals with the optimal fitness from the current population as the elite group.
(2) Definition of the dynamic interval: Determine the dynamic boundaries according to the distribution of the elite group in the *j*-th dimension. $a_{j}(t)$ is the minimum value of the elite group in the *j*-th dimension, and $b_{j}(t)$ is the maximum value of the elite group in the *j*-th dimension.
(3) Generation of opposition solutions: Combined with the random scaling coefficient k∈[0, 1], the mathematical expression of the opposition solution $x_{ij}^{*}(t)$ is

(24) $$x_{ij}^{*}(t)=k(a_{j}(t)+b_{j}(t))-x_{ij}(t),$$

where $x_{ij}(t)$ is the value of the current individual in the *j*-th dimension. Figure 2 is the schematic diagram of this strategy.

After the EOBL strategy, the fitness function value of the corresponding individual is computed. By contrasting the fitness function values between the current individual and the optimized individual, the one with a better fitness value is selected as the initial population individual. The following formula is used to optimize randomly initialized individuals:

(25) $$X_{i}=\left\{\begin{array}{ll} {\bar{X}}_{i}, & f({\bar{X}}_{i})<f(X_{i})\\ X_{i}, & \mathrm{else} \end{array}\right..$$

### 4.2. Adaptive Weight Mechanism

**Figure 3:**
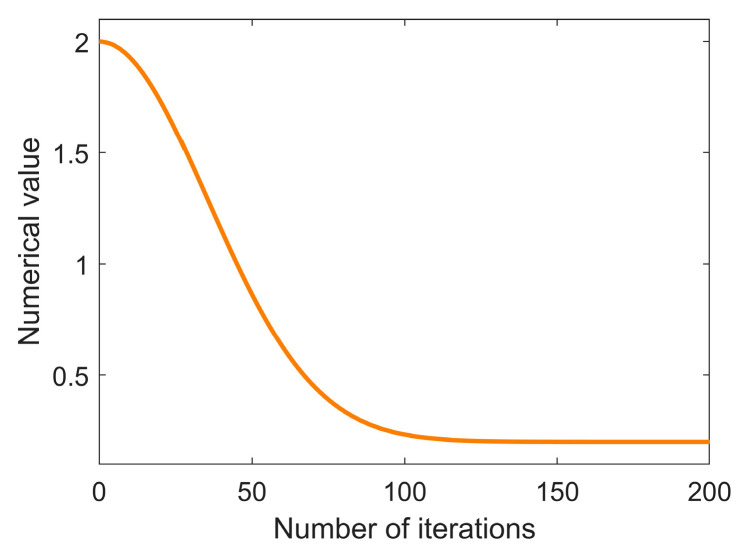
The nonlinear variation curve of the adaptive weight.

To balance global and local search, enhance BES convergence speed and accuracy, and prevent premature convergence, an adaptive nonlinear weighting mechanism based on exponential power attenuation is introduced [56]. Additionally, the fixed parameter controlling the spiral trajectory is modified. This mechanism dynamically adjusts the weight factor: maintaining a high value initially for stronger global exploration, accelerating decay in the middle phase to balance exploration and exploitation, and gradually slowing decay in the final stage to refine local search accuracy. The specific formula is as follows:

(26) $$\omega (t)=\omega_{\min}+(\omega_{\max}-\omega_{\min})\exp(-{\left(\frac{\eta \cdot t}{T}\right)}^{\mu }),$$

where t represents the current number of iterations, and T is the maximum number of iterations. $\omega_{\max}$ and $\omega_{\min}$ are the maximum and minimum values of the weight, respectively, with values of 2 and 0.2, respectively. η controls the decay rate, with a value of 4. μ adjusts the shape of the decay curve, and its value is 2. The curve of the adaptive nonlinear weight is shown in Figure 3.

Thus, the formula for the latest selection phase of the algorithm is

(27) $$X_{i,new}=X_{\mathrm{best}}+\omega (t)\cdot r(X_{\mathrm{mean}}-X_{i}).$$

### 4.3. Block-Based Elite-Guided Differential Mutation Strategy

In this paper, a block-based elite-guided differential mutation strategy (BEDMS) is designed. Its core objective is to balance the global exploration and local exploitation capabilities of the algorithm through the collaborative mechanism of dynamic block competition and elite guidance, effectively addressing issues such as the tendency of the traditional bald eagle search algorithm to get trapped in local optima and its insufficient convergence speed in high-dimensional nonlinear scenarios like path planning. This strategy includes the following three crucial stages [57]:

(1) Dynamic blocking mechanism

Let the population size be N. In the *t*-th iteration, the population is divided into two competitive subgroups through the index permutation function idx=randperm(N):

(28) $$\left\{\begin{array}{l} X_{1}=\left\{X_{idx(k)}|\;k=1,\ldots ,\;\left\lfloor N/2\right\rfloor \right\}\\ X_{2}=\left\{X_{idx(k)}|\;k=\left\lfloor N/2\right\rfloor +1,\ldots ,2\;\left\lfloor N/2\right\rfloor \right\} \end{array}\right..$$

(2) Generation of differential perturbation

Construct a directional mutation vector by utilizing the differences between the subgroups, and introduce the convergence factor F for nonlinear modulation:

(29) $$\Delta =F\cdot (X_{1}-X_{2}).$$

This formula ensures that the perturbation maintains high-intensity exploration in the early stage of the iteration t≤T and shifts to fine exploitation in the later stage t→T.

(3) Elite-guided mutation injection

Inject the perturbation into the first half of the population while retaining the information of the current optimal solution $X_{\mathrm{best}}$:

(30) $$X_{new}^{\left(1:\left\lfloor N/2\right\rfloor \right)}=X_{\mathrm{best}}+0.5\cdot (X^{\left(1:\left\lfloor N/2\right\rfloor \right)}+\Delta ).$$

This operation is equivalent to constructing a dynamically shrinking search hypercube in the neighborhood of the elite solution. Its volume is controlled by the convergence factor F, and the mathematical description is as follows:

(31) $$X_{\mathrm{mutant}}=\left\{\begin{array}{ll} X_{\mathrm{best}}+0.5\cdot (X+F\cdot (X_{1}-X_{2})), & \mathrm{if}\;rand<q\\ X, & \mathrm{otherwise} \end{array}\right..$$

**Figure 4:**
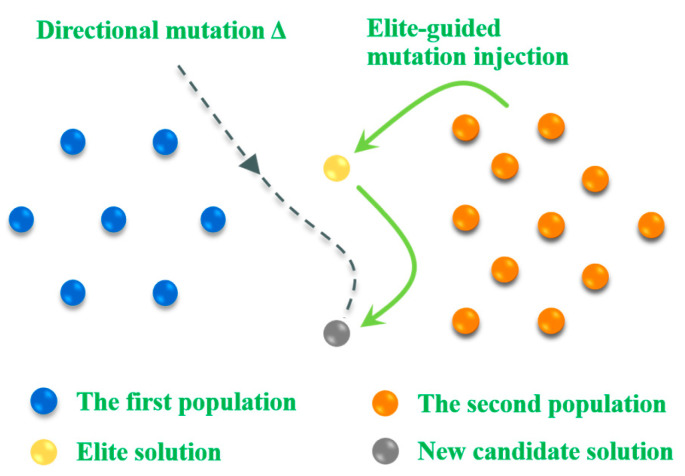
Block-based elite-guided differential mutation.

The principle of this strategy is shown in Figure 4.

**Figure 5:**
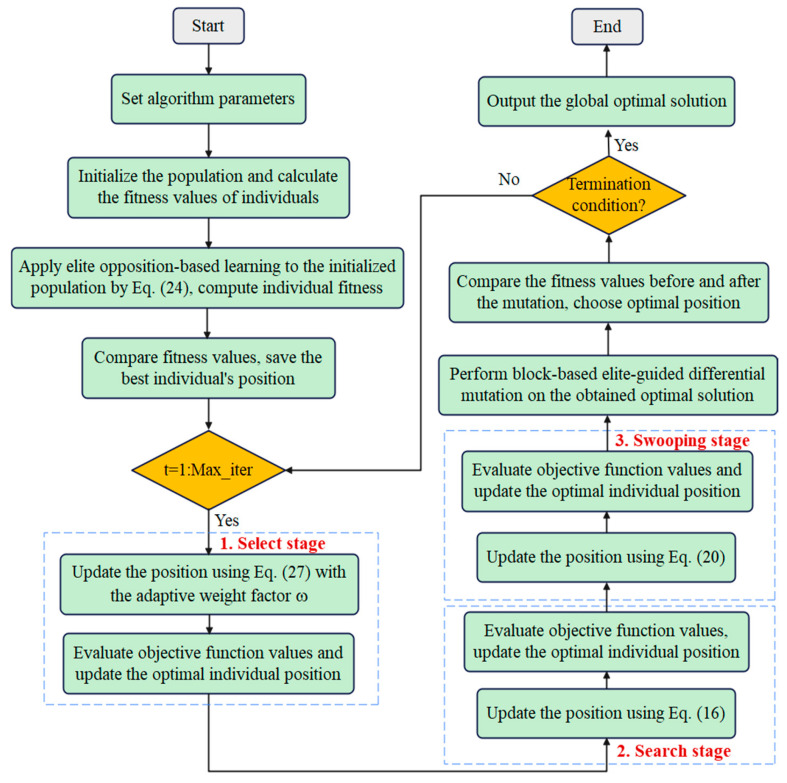
The flowchart of the EAB-BES algorithm.

In order to demonstrate the working mechanism of the EAB-BES algorithm more clearly, a flowchart of the EAB-BES algorithm integrating these strategies and mechanisms was drawn, as shown in Figure 5. The pseudocode of the EAB-BES algorithm is shown in Algorithm 2.
**Algorithm 2:** The pseudo-code of EAB-BES**Input**: *N* (population size), *D* (dimension), *Max_iter* (max iterations), *up*, *lb* (bounds) 1. Initialize population $X_{i}$ randomly and calculate fitness $f(X_{i})$ 2. Apply elite opposition-based learning on individual population by Equation (24)  and calculate new $f(X_{i})$ 3. Update $X_{i}$ with better individuals Compare the fitness values before and  after optimization, select the individual position $X_{best}$ with the best fitness  value as the optimal position. 4. **while** (t ≤ *Max_iter*) 5.  Update adaptive weight ω(t) by Equation (26) 6.     **for** *i* = 1 to *N*        **Select stage** 7.        Update individual position $X_{i}$ by Equation (27)        **Search stage** 8.        Update individual position $X_{i}$ by Equation (16)        **Swooping stage** 9.        Update individual position $X_{i}$ by Equation (20) 10.     Apply a block-based elite-guided differential mutation strategy        to the current optimal solution. 11.        **if**
$f(Xnew)<f(X_{i})$ 12.          $X_{i}=X_{new}$ 13.        **end if** 14.        **if** f(Xnew)<f(Xbest) 15.          Xbest=Xnew 16.        **end if** 17.      **end for** 18.      t = t + 1 19. **end while** 20. **Output** $X_{\mathrm{best}}$

### 4.4. Computational Complexity Analysis

The BES exhibits a time complexity of O(T⋅N⋅F⋅D) (where N,D,T and F represent population size, dimensionality, iterations, and fitness evaluation cost) and space complexity O(N⋅D). The EAB-BES integrates three strategies: elite opposition-based learning (doubling fitness evaluations per iteration to O(2N⋅F⋅D)), difference mutation (adding O(N⋅D) step-size calculations), and adaptive weighting (O(N) per iteration). While preserving the asymptotic time complexity O(T⋅N⋅F⋅D) and space complexity O(N⋅D) (storing population and opposition solutions). Although the time complexity of EAB-BES is still O(T⋅N⋅F⋅D), the number of fitness assessments has increased, and the cost of location updates and parameter calculations has slightly increased. The spatial complexity remains unchanged, with only a small amount of parameter storage added without changing the size. EAB-BES improves performance but also increases some computational costs.

## 5. Experimental Results and Discussion

### 5.1. Experimental Setup

This study compared nine algorithms with EAB-BES, including the classic particle swarm optimization algorithm [58] (PSO), differential evolution [59] (DE), teaching-learning-based optimization [60] (TLBO), the emerging excellent algorithms—slime mold algorithm [61] (SMA) and sparrow search algorithm [62] (SSA)—and the improved high-performance algorithms—teaching learning slime mold algorithm [63] (TLSMA), chaotic sparrow search algorithm (CSSA) [64], bald eagle search algorithm [22] (BES), and modified bald eagle search algorithm (mBES) [65]. To test the performance and effectiveness of EAB-BES, these algorithms were applied to solve the path planning problem of 3D UAVs in complex urban scenes. In each experiment, each algorithm was configured to perform 200 iterations with a population size of 100, and the entire process was independently run and repeated 20 times. The dimension is set to 30. The experiment was conducted in the MATLAB R2019(a) environment. The desktop computer adopted an Intel (R) Core (TM) i7-97000 CPU, featuring a 3.00 GHz main frequency, 64-bit operating system, and 16 GB RAM. Table 1 presents the parameter settings for various algorithms.

### 5.2. UAV Flight Environment Description

To evaluate the performance, effectiveness, and feasibility of the EAB-BES algorithm, this paper applies it along with other comparative algorithms to solve the 3D path planning problem for UAVs in urban environments. In this study, six types of urban environments are designed, where cylinders and hemispheres are used to represent high-rise buildings and low-rise structures, respectively. The complexity of the obstacles, including their number, height, and breadth, increases progressively, leading to a corresponding rise in the difficulty level.

In Environment 1 and Environment 2, the coordinates of the starting and goal points are identical. These environments are relatively simple, characterized by a small number of buildings and smaller radii. And these tests are the flight conditions of different algorithms in low spaces. The primary difference is that Environment 2 features an increased number of buildings and larger radii. The heights of high-rise buildings remain consistent across Environment 1 to Environment 3, whereas only the heights of low-rise buildings vary. The difficulty level rises sharply from Environment 4 to Environment 6, with significant increases in the number, height, radius, and density of both high-rise and low-rise buildings. Among these, the tallest building exceeds 9000 m in height, while the largest radius reaches 2000 m. The building positions vary across the six environments from Environment 1 to Environment 6.

In Environment 1 to Environment 4, the UAV’s path primarily follows a diagonal trajectory. In Environment 1 and Environment 2, the starting and ending points share the same altitude, enabling parallel flight. In Environment 3, the ending point is at a lower altitude than the starting point, resulting in a descending flight path. In Environment 4, the ending point is at a higher altitude than the starting point, resulting in an ascending flight path. In Environment 5 and Environment 6, the UAV’s path primarily follows the coordinate axes, with a descending flight in Environment 5 and an ascending flight in Environment 6.

In the 3D flight diagram, the start is marked with a red dot, and the goal with a five-pointed star. Altitude is encoded using a color gradient: dark blue for low altitudes, transitioning from cyan–green–yellow to red for high altitudes. This design visually emphasizes altitude variations and terrain undulations in the study area. The detailed configurations of the six 3D test environments are presented in Table 2.

### 5.3. Analysis of Experimental Results

**Figure 6:**
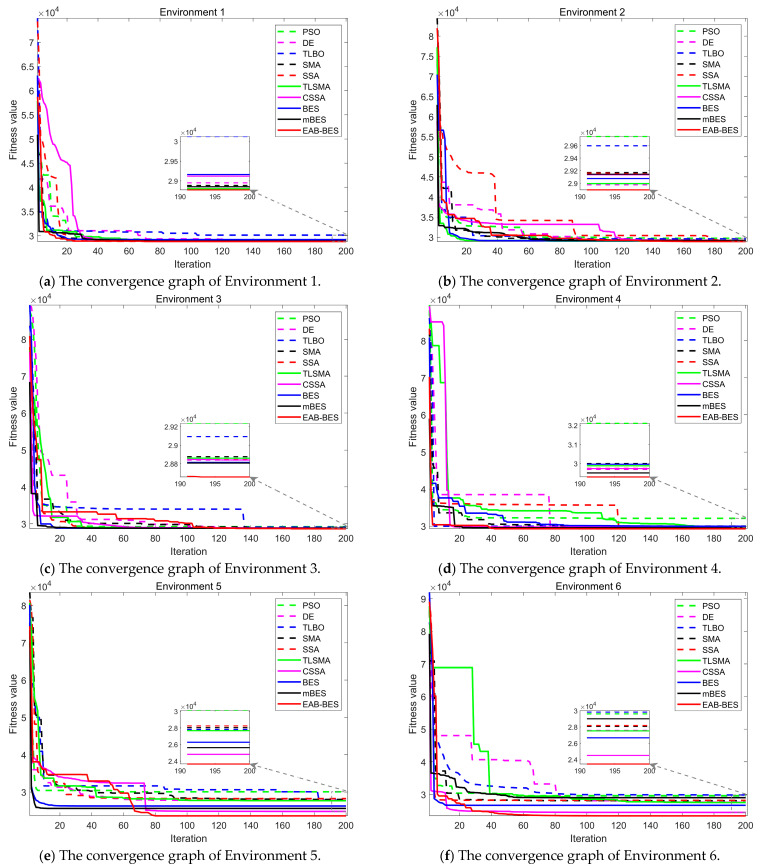
The convergence curves of comparative algorithms for all environments.

Figure 6 shows the convergence curves of the best fitness values for the 3D path planning of the UAV in six environments. After 200 iterations, each algorithm can converge normally. However, in all environments, the EAB-BES algorithm has a faster convergence speed and can achieve the minimum precision value. It can timely escape from local optimal traps, has strong convergence stability, can find the optimal solution to the problem, and search for the shortest planned path.

Figure 6a,b illustrates the convergence situations of different algorithms in Environments 1 and 2. Since the first two environments are relatively simple and do not impose high requirements on the algorithm performance, all algorithms can converge to a relatively good value. At the initial stage of iteration, the fitness values obtained by the algorithms are relatively large, while the convergence values are relatively small, resulting in a significant data gap. In the later stage, it is difficult to distinguish the differences in the convergence values of different algorithms. Therefore, a small window was intentionally opened in the convergence graph. Although in some individual scenarios, the fitness values of UAV path planning did not differ significantly, it can be clearly observed in the small window that the EAB-BES still achieved the minimum fitness convergence value, with faster convergence speed and the strongest performance.

In Environments 3–6, corresponding to Figure 6c–f, it can be more intuitively seen that compared with the comparison algorithms, within the same number of iterations, the EAB-BES algorithm shows a faster trend of approaching the optimal solution. The fitness value fluctuates minimally during the convergence process, indicating good stability. The EAB-BES algorithm has extremely high curve smoothness. In contrast, the fluctuations of the DE and TLSMA algorithms are relatively large. Under the same test environment, the difference in the final convergence fitness values between the EAB-BES algorithm and other algorithms is obvious. The fitness value of the EAB-BES algorithm is significantly the lowest. The performance of BES is also good. Other comparison algorithms, such as PSO, TLBO, and SSA, may fall into local optima and exhibit premature convergence.

In Figure 6e, after the fitness value of the EAB-BES algorithm reaches a certain level at the initial stage of iteration, it successfully decreases to near the minimum value after 50 more generations of further search, effectively avoiding the premature convergence phenomenon and ensuring the acquisition of the global optimal solution. Relying on its unique differential mutation strategy based on block-based elite guidance, it successfully escapes from the local optimal region in subsequent iterations, and the fitness value continues to decline and finally converges near the global optimum. This characteristic enables the algorithm to have stronger robustness in complex solution spaces and greatly improves the probability of finding the true optimal solution. The comparison of the convergence curves proves the superiority of the EAB-BES in solving the UAV path planning problem, which can obtain better results and demonstrate better performance.

Table 3 presents a detailed numerical comparison of all algorithms across different urban environments, including the best value, worst value, mean value, and standard deviation. From the table, it can be observed that EAB-BES outperforms other algorithms in most numerical comparisons. It achieves significantly lower values in terms of the best value, worst value, and mean value. In terms of standard deviation, EAB-BES is less volatile than other algorithms in most scenarios, highlighting the reliable performance of this algorithm under different operating conditions. DE also shows certain advantages in the standard deviation index. Similarly to the results shown in the convergence curves, the advantage of EAB-BES in Environment 1 and Environment 2 is not as pronounced but still superior to other algorithms, which is attributed to the simplicity of the building structures in these environments.

In Environment 3 to Environment 6, due to the increased density, height, and radius of both high-rise and low-rise buildings in the urban environment, as well as the adjustment of the UAV’s starting and goal point altitudes, the UAV’s flight path involves both descending and ascending trajectories. As a result, the shortcomings of the comparative algorithms become evident, as they tend to fall into premature convergence, experience reduced accuracy, and fail to meet the requirements for optimal path planning. TLSMA, CSSA, and mBES, these excellent improved algorithms, also perform well in terms of optimal values and can generally find suboptimal solutions.

In contrast, the EAB-BES algorithm, enhanced by elite opposition-based learning, adaptive weighting, and a block-based elite-guided differential mutation strategy, overcomes the tendency to fall into local optima. It significantly improves accuracy and convergence speed, effectively balancing local and global search capabilities. Therefore, EAB-BES can find superior solutions compared to other algorithms, plan the shortest routes, and efficiently solve the 3D UAV path planning problem in urban environments.

**Figure 7:**
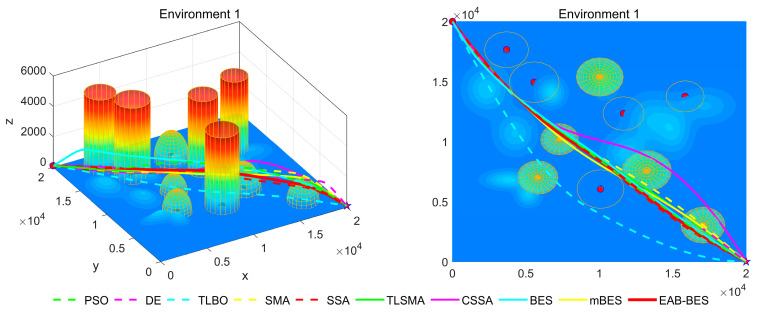
3D path stereogram and top view of Environment 1.

**Figure 8:**
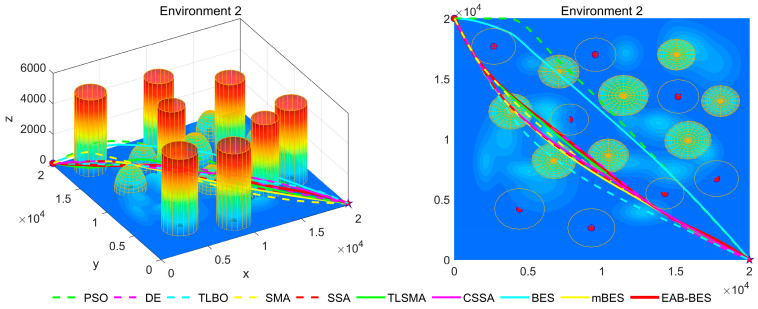
3D path stereogram and top view of Environment 2.

**Figure 9:**
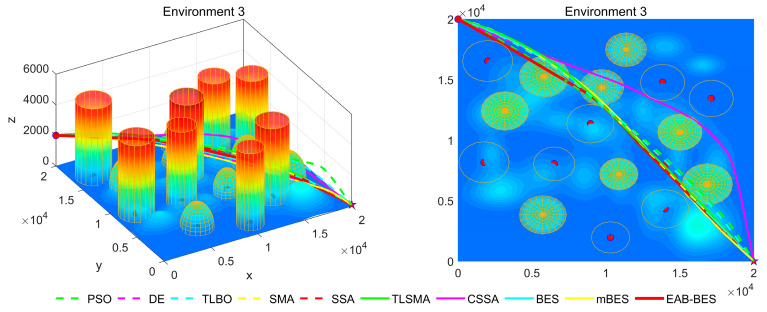
3D path stereogram and top view of Environment 3.

**Figure 10:**
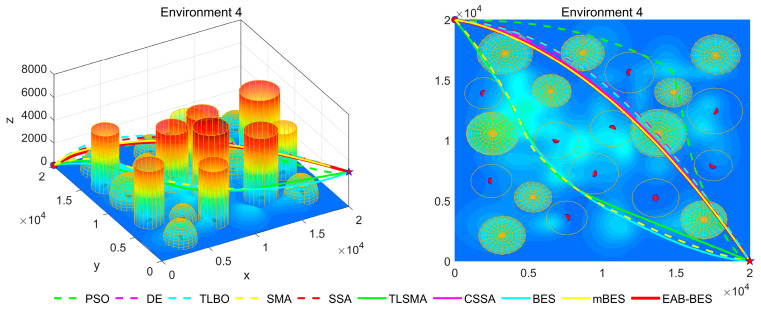
3D path stereogram and top view of Environment 4.

**Figure 11:**
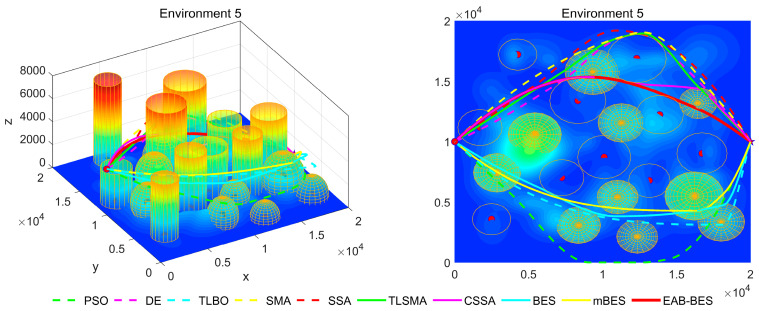
3D path stereogram and top view of Environment 5.

**Figure 12:**
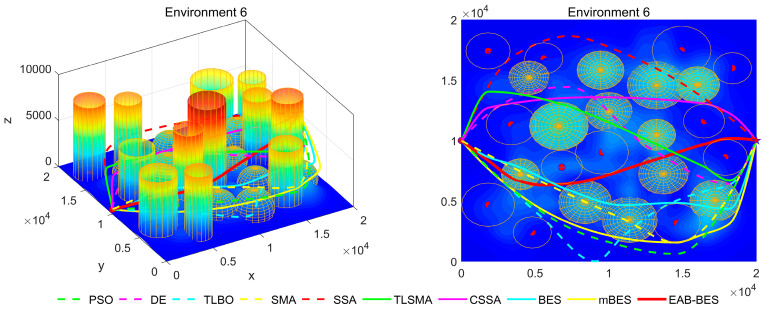
3D path stereogram and top view of Environment 6.

Figure 7, Figure 8, Figure 9, Figure 10, Figure 11 and Figure 12 present the 3D stereograms and top views of different algorithms for addressing the UAV path planning problem in urban environments. In these figures, the EAB-BES algorithm exhibits remarkable advantages compared with comparative algorithms such as PSO, DE, and TLBO. In Environments 1 and 2, although the complexity of obstacles is low, EAB-BES still plans shorter and smoother paths. As the complexity of the environment increases, for example, in Environments 3–6, the obstacle avoidance ability and path optimization advantages of EAB-BES become more apparent as the density, height, and distribution complexity of high-rise and low-rise buildings increase. Under constraint compliance, its planned path accurately avoids various obstacles, including high-rise buildings (columnar) and low-rise buildings (hemispherical), eliminating redundant detouring. Compared with algorithms like PSO and SSA, it features shorter path length and better continuity. This performance benefits from the elite opposition-based learning, adaptive weights, and block-based elite-guided differential mutation strategies integrated into EAB-BES, enabling effective balance between local and global search capabilities in complex urban 3D environments. It thus effectively verifies the algorithm’s excellent performance, as well as its efficiency and reliability in solving the 3D UAV path planning problem across diverse and complex scenarios. In high-difficulty and high-density scenarios such as Environment 4, 5, and 6, the CSSA and mBES algorithms can also plan shorter collision-free paths, which is due to the advanced strategies within the improved algorithms.

In Table 4, we conducted an ablation experiment on the four key parameters (η, $\omega_{\max}$, $\omega_{\min}$, μ) of the EAB-BES algorithm to verify their impact on performance. The ablation experiment was conducted in the most challenging Environment 6 to verify the generalization of the proposed algorithm, and the Mean, Best, Worst, and Std were reported. The results show that the original parameter configuration (η = 4, $\omega_{\max}$ = 2, $\omega_{\min}$ = 0.2, μ = 2) achieved the best performance in all indicators, indicating that this configuration achieved a good balance between accuracy and stability.

From the comparison, it can be observed that increasing the η value (such as η = 5) or adjusting $\omega_{\max}$ (such as setting it to 1.5 or 2.5) will cause a slight decrease in the average fitness and the best value. At the same time, reducing or increasing the $\omega_{\min}$ and μ values will also weaken the convergence effect and robustness of the algorithm to a certain extent. This result further verifies that the parameter configuration proposed in this paper has good adaptability and adjustment ability, which can not only accelerate convergence but also effectively prevent falling into local optimality.

**Figure 13:**
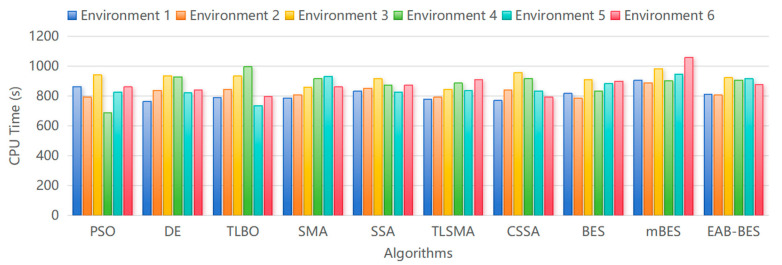
Comparison of CPU running time for different algorithms.

As illustrated in Figure 13, the CPU time of the EAB-BES algorithm is moderately higher than that of simpler algorithms such as DE, PSO, and TLBO, particularly in complex urban environments like Environments 4, 5, and 6. For instance, in Environment 6, EAB-BES requires approximately 877.9 s, compared to 840.4 s for DE—an increase of about 4.5%. This additional computational cost is primarily attributed to the integration of multiple strategies in EAB-BES, which significantly enhance global search capability and path accuracy.

Nevertheless, this trade-off proves to be well justified. Despite the increased runtime, EAB-BES achieves substantial improvements in overall performance metrics. In the same environment, the best path cost is reduced by 23.2%, and the standard deviation is lowered by 30.2%, indicating improved robustness and consistency. Among advanced algorithms like BES and mBES, EAB-BES also attains the shortest CPU time, reflecting its enhanced search efficiency.

Furthermore, the average CPU time of EAB-BES across six environments remains under 950 s, which is acceptable for offline preflight planning or near real-time route updates in practical UAV missions. In mission-critical scenarios where navigation precision, obstacle avoidance, and environmental adaptability are essential, the performance gains of EAB-BES outweigh the moderate increase in computational load.

Therefore, EAB-BES strikes a favorable balance between optimization performance and computational efficiency, making it a reliable and scalable solution for 3D UAV path planning tasks in complex urban environments.

### 5.4. Statistical Hypothesis Test

To validate the scientific validity and credibility of the proposed algorithm and analyze its performance advantages, statistical hypothesis tests were performed. The Wilcoxon rank-sum test quantifies significant differences between paired algorithms using *p*-values [66]. The Friedman test compares performance rankings across multiple environments to assess macro-level algorithmic superiority [67].

From the statistical test results, Table 5 presents the *p*-values from the Wilcoxon rank-sum test. The analysis reveals that in the vast majority of environments, the *p*-values for comparisons between EAB-BES and algorithms such as PSO, DE, and TLBO are significantly lower than the conventional significance level of 0.05. This strongly demonstrates that the performance differences between EAB-BES and the comparative algorithms are statistically significant. Although a few pairwise comparisons do not reach significance, the overall test results robustly support the conclusion that EAB-BES exhibits more reliable performance advantages in UAV path planning tasks compared to other algorithms, validating its effectiveness and superiority.

Table 6 shows the results of the Friedman test. The data indicate that the EAB-BES algorithm ranks first from Environment 1 to Environment 6, with significantly lower average scores than comparative algorithms. This demonstrates its superior comprehensive performance across multiple urban environments.

To enrich statistical tests, we calculated Cohen’s d effect sizes to assess the practical significance of the observed differences. Unlike *p*-values, which indicate statistical significance, Cohen’s d quantifies the magnitude of the difference between two groups in standardized units. Typically, values of d > 0.2, 0.5, and 0.8 are interpreted as small, medium, and large effects, respectively.

As shown in Table 7, EAB-BES demonstrates moderate to large effect sizes (d > 0.8) in most scenarios, especially against PSO, SMA, and mBES. For example, in Environment 5, EAB-BES exhibits a very large effect (2.22) when compared with PSO, confirming its substantial improvement. These results indicate that the performance advantage of EAB-BES is not only statistically significant but also practically meaningful in real-world UAV path planning applications.

**Figure 14:**
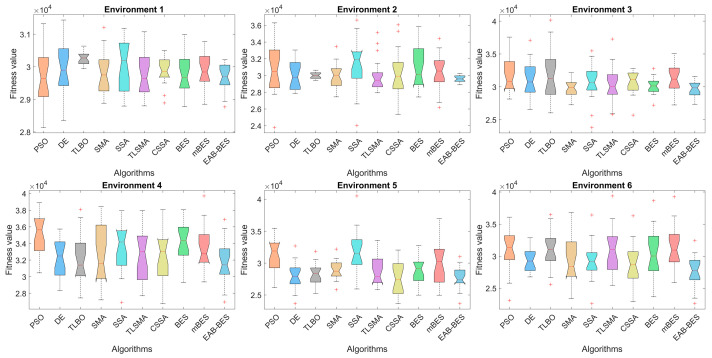
Box plot comparison of different algorithms in all environments.

Box plots visualize performance distributions of algorithms in 3D UAV path planning for urban environments. The median represents average planning accuracy, the interquartile range (IQR) measures fitness value dispersion (stability), and outliers indicate abnormal fluctuations during execution [68]. Figure 14 presents box plots that compare the performance of algorithms across six urban environments in terms of planning accuracy (median), stability (IQR), and robustness (outliers). EAB-BES consistently achieves lower medians, smaller box heights, and fewer outliers than most comparative algorithms.

In Environments 1 to 6, EAB-BES demonstrates superior fitness values, reduced variability, and minimal abnormal fluctuations. Notably, its box plots are markedly compact and positioned lower than others, reflecting high accuracy and strong stability. For example, in Environments 1, 2 and 3, EAB-BES exhibits almost no outliers, while PSO and DE show pronounced fluctuations. EAB-BES achieves a favorable balance of precision, stability, and reliability in repeated runs, confirming its effectiveness for robust 3D UAV path planning in complex urban scenarios.

### 5.5. Discussion

The experimental results across six heterogeneous environments demonstrate the superior performance of the EAB-BES algorithm in 3D UAV path planning. Compared to the BES, EAB-BES achieved significant improvements in core metrics: a 5.2% reduction in Best (path length), a 3.8% optimization in Mean performance, and a 41.2% enhancement in robustness (Std) on average. In Environment 5 with stringent dynamic constraints, EAB-BES reduced the best value by 10.03% and optimized Std by 31.6%, highlighting its adaptability to complex scenarios.

When compared to mainstream algorithms, EAB-BES consistently outperformed competitors in multiple dimensions. For instance, it achieved 20.8% lower best values than PSO in Environment 6 and a 14.6% improvement over TLSMA in Environment 5. In terms of convergence stability, EAB-BES maintained the lowest Mean values across all environments, with a 10.0% optimization over PSO in Environment 4. While TLBO exhibited the lowest Std (195.30) in Environment 1 and DE achieved minimal Std (1705.75) in Environment 6, EAB-BES demonstrated balanced robustness, particularly excelling in dynamic environments (e.g., 344.93 Std in Environment 2, 80.2% lower than DE).

Statistical analyses further validate these findings. The Wilcoxon rank-sum test showed that the majority of *p*-values were significantly below 0.05, confirming the statistical significance of EAB-BES’s improvements. The Friedman test ranked EAB-BES first overall, underscoring its comprehensive superiority. These results can be attributed to the algorithm’s integration of elite opposition-based learning, adaptive weighting mechanisms, and block-based elite-guided differential mutation, which collectively enhance exploration–exploitation balance and adaptability to high-density urban environments.

These results underscore the effectiveness of EAB-BES in harmonizing exploration–exploitation trade-offs, especially under strict constraints. The EAB-BES’s ability to minimize path length while ensuring stability positions it as a robust solution for real-world UAV applications, particularly in complex urban landscapes requiring high precision and adaptability.

## 6. Conclusions

This study introduces a multi-strategy enhanced bald eagle search algorithm (EAB-BES) to address critical challenges in 3D UAV path planning within complex urban environments. By integrating elite opposition-based learning to broaden solution space exploration, adaptive weight mechanisms to dynamically balance global–local search trade-offs, and block-based elite-guided differential mutation to refine local optimization precision, EAB-BES systematically enhances the capabilities of the conventional BES algorithm across three key dimensions. Extensive experiments conducted in six high-density scenarios with mixed high-rise and low-altitude obstacles validate its superiority in generating collision-free paths, achieving the shortest path length, highest smoothness, and fastest convergence speed among comparative algorithms. Statistical analyses, including the Wilcoxon rank-sum, Friedman test, box plot evaluations, and Cohen’s d effect size analysis, further confirm its robustness in avoiding local optima, the practical significance of performance gains, and its stable behavior under stringent spatial constraints. An ablation study further verifies the robustness of key parameter settings, demonstrating balanced convergence and generalization ability under varying configurations. Computational efficiency analysis reveals that the marginal increase in CPU time is well compensated by notable gains in path quality and robustness, making EAB-BES suitable for near real-time or offline mission planning scenarios. These comparative results demonstrate that EAB-BES consistently identifies superior solutions, efficiently resolving 3D path planning challenges in urban environments and offering practical benefits for urban surveillance, emergency response, and logistics applications.

Future research will focus on integrating EAB-BES with reinforcement learning for real-time dynamic obstacle avoidance [69,70,71] and extending the framework to multi-UAV collaborative systems in ultra-high-density cities for decentralized path coordination [72,73]. Practical planning of low-cost UAVs in real scenarios can also be explored to improve adaptability across heterogeneous environments, especially in enhancing model deployment and data transmission efficiency within communication-constrained UAV clusters [74,75,76,77].

## Figures and Tables

**Table 1:** The parameter settings of algorithms.

Algorithms	Parameter Settings
PSO	w=0.9, $C_{1}=C_{2}=2$
DE	F=0.5, $C_{r}=0.9$
TLBO	TF∈{1,2}, $r_{i}\in [0,1]$
SMA	z=0.03
SSA	PD=0.2PopSize, SD=0.1PopSize, ST=0.8
TLSMA	TF∈{1,2}, $r_{i}\in [0,1]$, z=0.03
CSSA	PD=0.2, SD∈[0.1, 0.2], ST=0.8, $\mu_{log}=3.99$, $w_{0}=0.9$, c=0.9
BES	α=2, a=10, R=1.5, $c_{1}=c_{2}=2$, r∈[0, 1]
mBES	α=2, a=10, R=1.5, $c_{1}=c_{2}=2$, r∈[0, 1]
EAB-BES	$\begin{array}{c} \alpha =2,\;a=10,\;R=1.5,\;c_{1}=c_{2}=2,\;r\in [0,\;1],\;k\in [0,\;1],\\ \omega_{max}=2,\;\omega_{min}=0.2,\;\eta =4,\;\mu =2,\;\beta =1.5 \end{array}$

**Table 2:** Details of different 3D test environments.

Environments	Start Point	Goal Point	Position of High-Rise Buildings		Position of Low-Rise Buildings	
			Projection Coordinate	Height, Radius	Projection Coordinate	Radius
Environment 1	(0, 20000, 100)	(20000, 0, 100)	(15845, 13757, 0)	5000, 1300	(7283, 10186, 0)	1300
			(11655, 12328, 0)	5000, 1400	(5794, 6991, 0)	1400
			(3683, 17649, 0)	5000, 1500	(17063, 3049, 0)	1500
			(10083, 6039, 0)	5000, 1600	(10034, 15375, 0)	1600
			(5567, 14937, 0)	5000, 1700	(13216, 7566, 0)	1700
Environment 2	(0, 20000, 100)	(20000, 0, 100)	(14269, 5484, 0)	5000, 1300	(18038, 13163, 0)	1300
			(15174, 13534, 0)	5000, 1400	(7064, 15587, 0)	1400
			(2667, 17685, 0)	5000, 1500	(3823, 12278, 0)	1500
			(9284, 2633, 0)	5000, 1600	(15831, 9895, 0)	1600
			(4422, 4209, 0)	5000, 1700	(11449, 13609, 0)	1700
			(7810, 11614, 0)	5000, 1300	(15061, 17010, 0)	1300
			(9561, 17000, 0)	5000, 1400	(10435, 8614, 0)	1400
			(17766, 6717, 0)	5000, 1500	(6732, 8174, 0)	1500
Environment 3	(0, 20000, 2000)	(20000, 0, 100)	(10312, 1963, 0)	5000, 1300	(10874, 7195, 0)	1300
			(6500, 8040, 0)	5000, 1400	(11371, 17484, 0)	1400
			(13832, 14768, 0)	5000, 1500	(9714, 14344, 0)	1500
			(8948, 11361, 0)	5000, 1600	(3164, 12413, 0)	1600
			(2002, 16532, 0)	5000, 1700	(16851, 6353, 0)	1700
			(17122, 13444, 0)	5000, 1500	(15003, 10659, 0)	1500
			(14002, 4327, 0)	5000, 1600	(5727, 3857, 0)	1600
			(1757, 8175, 0)	5000, 1700	(5818, 15221, 0)	1700
Environment 4	(0, 20000, 100)	(20000, 0, 3000)	(9415, 7258, 0)	6000, 1700	(5301, 5310, 0)	1300
			(7590, 3590, 0)	5000, 1400	(6504, 14073, 0)	1400
			(6869, 10045, 0)	5000, 1500	(8681, 17245, 0)	1500
			(13617, 5224, 0)	6000, 1600	(16878, 3426, 0)	1600
			(17634, 12447, 0)	5000, 1900	(17673, 17059, 0)	1700
			(17522, 7928, 0)	4000, 1300	(2560, 10564, 0)	1800
			(2479, 6642, 0)	5000, 1400	(3405, 17190, 0)	1900
			(10672, 10981, 0)	5000, 1500	(13778, 10590, 0)	2000
			(1957, 13903, 0)	5000, 1300	(3204, 2153, 0)	1600
			(11870, 15626, 0)	3000, 1700	(14819, 14020, 0)	1300
Environment 5	(0, 10000, 4000)	(20000, 10000, 500)	(12307, 16887, 0)	5000, 2000	(18011, 3362, 0)	1600
			(13862, 12235, 0)	3000, 1600	(12346, 2149, 0)	1400
			(7236, 7024, 0)	5000, 1500	(11007, 5410, 0)	1400
			(8325, 13398, 0)	6000, 1900	(5412, 10606, 0)	1800
			(16720, 9031, 0)	5000, 1700	(8362, 3073, 0)	1500
			(4259, 17214, 0)	7000, 1300	(11286, 11556, 0)	1600
			(13161, 6781, 0)	5000, 1400	(2859, 7427, 0)	1600
			(1705, 11170, 0)	3000, 1500	(9344, 15785, 0)	1900
			(10107, 8725, 0)	4000, 1800	(16204, 5497, 0)	2000
			(2487, 3581, 0)	5000, 1300	(16424, 13188, 0)	1300
Environment 6	(0, 10000, 500)	(20000, 10000, 5000)	(18380, 15991, 0)	6000, 1300	(13683, 7254, 0)	1700
			(6808, 7803, 0)	7000, 1400	(11402, 3462, 0)	1900
			(1808, 17415, 0)	8000, 1500	(16021, 14619, 0)	1500
			(14600, 3189, 0)	6000, 1600	(9434, 15827, 0)	1600
			(3376, 11338, 0)	5000, 1700	(17182, 5046, 0)	1700
			(9536, 9027, 0)	9000, 1800	(4004, 7203, 0)	1300
			(4807, 2406, 0)	7000, 1300	(4586, 15201, 0)	1400
			(16451, 11611, 0)	7000, 1400	(6609, 11204, 0)	2000
			(14932, 17467, 0)	6000, 2000	(10002, 12396, 0)	1600
			(17894, 8620, 0)	8000, 1500	(13238, 10476, 0)	1300
			(5580, 16794, 0)	7000, 1300	(13236, 14589, 0)	2000
			(1817, 4687, 0)	6000, 1800	(8378, 4910, 0)	1800

**Table 3:** Experimental results in different environments.

Environments	Indicators	PSO	DE	TLBO	SMA	SSA	TLSMA	CSSA	BES	mBES	EAB-BES
Environment 1	Best	28869.11	28949.14	30123.47	28883.30	28795.45	28800.05	29118.29	29159.93	28850.08	**28770.17**
	Worst	30469.13	30654.39	30634.86	30655.91	30563.58	30406.99	30225.88	30557.60	30406.79	**30132.65**
	Mean	29710.29	29982.09	30261.97	29808.36	30022.42	29742.09	29899.73	29755.62	29911.28	**29700.06**
	Std	817.64	776.74	**195.30**	640.50	746.99	610.83	377.84	594.27	455.77	352.08
Environment 2	Best	29743.19	28974.45	29595.99	29170.65	29150.50	28994.96	29133.76	29074.87	29143.47	**28894.00**
	Worst	36292.54	33049.17	30575.89	33467.23	35039.12	35133.74	35300.77	34852.18	34411.81	**30263.82**
	Mean	30521.53	29978.62	29986.98	29787.02	31219.78	29757.19	30015.95	30771.36	30536.50	**29627.45**
	Std	2883.92	1738.53	364.18	1407.49	3001.44	1627.21	2605.28	2511.77	2041.02	**344.93**
Environment 3	Best	29235.86	28839.45	29092.15	28875.64	28845.19	28859.34	28840.61	28813.94	28806.88	**28655.60**
	Worst	35241.53	34209.93	40172.20	31693.25	34665.93	34177.82	32800.18	31893.60	34526.54	**31591.01**
	Mean	31509.19	31131.98	31508.20	29733.19	30574.57	30232.44	30634.01	30103.94	31125.37	**29656.72**
	Std	2558.71	2631.29	3676.53	1366.88	2724.41	2611.79	1724.71	1197.91	2106.37	**1153.68**
Environment 4	Best	32104.04	29687.32	29976.24	30000.67	29744.95	29877.29	29733.23	29959.94	29505.62	**29290.53**
	Worst	38424.62	35745.37	36158.35	37890.77	36782.14	37978.71	38111.97	37146.94	37416.69	**34313.32**
	Mean	35056.72	32253.08	31881.71	32401.20	33685.82	32358.58	32321.66	34237.60	33229.82	**31549.43**
	Std	2364.48	**2178.09**	2780.87	3644.21	2842.43	2996.20	3200.11	2381.97	2637.40	2525.59
Environment 5	Best	30082.34	27732.18	27809.34	28026.70	28238.98	27665.37	24809.38	26264.92	25616.97	**23637.72**
	Worst	35473.50	30815.47	30577.27	30203.63	33814.24	31021.27	31224.73	32572.90	31914.31	**30146.74**
	Mean	31241.24	27911.56	28178.77	28937.20	31909.51	28714.51	**27684.43**	28937.92	29964.84	27746.38
	Std	2335.51	2058.33	1620.60	1506.93	3360.33	2425.74	2491.36	2123.46	3271.77	**1451.82**
Environment 6	Best	29602.36	27573.96	29819.96	28069.59	28173.17	27515.35	24501.48	26667.52	28998.63	**23445.14**
	Worst	34345.67	32744.86	33811.11	36694.06	32948.58	35868.66	32601.12	35241.69	35104.26	**30529.16**
	Mean	30828.08	29352.27	30905.74	29277.92	29135.11	30683.45	28758.66	30077.51	31314.01	**27643.72**
	Std	3336.11	**1705.75**	2899.16	3713.67	2967.11	3680.63	3215.22	3834.74	3401.45	2376.33

The optimal results are shown in bold.

**Table 4:** Comparison of EAB-BES parameter ablation experiments on Environment 6.

No.	η	$\omega_{max}$	$\omega_{min}$	μ	Mean	Best	Worst	Std
1	3	2	0.2	2	24501.48	32601.12	28758.66	3215.22
2	4	2	0.2	2	**23445.14**	**30529.16**	**27643.72**	**2376.33**
3	5	2	0.2	2	24611.30	33012.05	28910.54	3412.09
4	4	1.5	0.2	2	23733.58	31115.88	28124.88	2945.87
5	4	2.5	0.2	2	23889.41	31901.67	28257.42	3080.25
6	4	2	0.1	2	24123.65	31774.33	28511.42	3001.89
7	4	2	0.3	2	24345.56	31345.99	28402.66	2966.72
8	4	2	0.2	1.5	24089.22	31805.74	28683.91	3033.15
9	4	2	0.2	2.5	23492.36	30777.21	27665.28	2452.68

The optimal results are shown in bold.

**Table 5:** Wilcoxon rank-sum test results (*p*-values) in different environments.

Environments	EAB-BES VS								
	PSO	DE	TLBO	SMA	SSA	TLSMA	CSSA	BES	mBES
Environment 1	4.78 × 10^−4^	8.89 × 10^−5^	7.10 × 10^−5^	1.06 × 10^−3^	4.38 × 10^−13^	3.85 × 10^−4^	1.18 × 10^−7^	1.99 × 10^−2^	**8.60 × 10^−1^**
Environment 2	3.87 × 10^−11^	2.42 × 10^−3^	5.99 × 10^−5^	**1.32 × 10^−1^**	3.85 × 10^−7^	2.43 × 10^−6^	1.34 × 10^−9^	8.24 × 10^−4^	6.32 × 10^−3^
Environment 3	**1.33 × 10^−1^**	1.09 × 10^−2^	2.28 × 10^−6^	4.57 × 10^−3^	1.12 × 10^−2^	3.87 × 10^−2^	1.00 × 10^−8^	1.97 × 10^−4^	2.27 × 10^−4^
Environment 4	2.07 × 10^−6^	1.68 × 10^−9^	6.68 × 10^−3^	3.29 × 10^−4^	3.28 × 10^−6^	7.86 × 10^−5^	4.44 × 10^−3^	4.98 × 10^−10^	1.10 × 10^−8^
Environment 5	1.44 × 10^−14^	4.28 × 10^−12^	2.25 × 10^−12^	9.05 × 10^−14^	1.12 × 10^−12^	8.30 × 10^−13^	3.21 × 10^−14^	1.67 × 10^−9^	3.83 × 10^−10^
Environment 6	2.14 × 10^−9^	6.18 × 10^−9^	1.78 × 10^−13^	3.61 × 10^−2^	4.37 × 10^−2^	2.62 × 10^−3^	4.01 × 10^−2^	4.12 × 10^−2^	2.23 × 10^−5^

No significant differences are shown in bold.

**Table 6:** Friedman test results in different environments.

Environments	Indicators	PSO	DE	TLBO	SMA	SSA	TLSMA	CSSA	BES	mBES	EAB-BES
Environment 1	Mean score	4.8	4.7	8.0	5.4	5.5	5.1	5.3	6.2	5.0	3.9
	Rank	3	2	10	7	8	5	6	9	4	1
Environment 2	Mean score	8.0	5.7	6.1	3.7	6.8	4.0	6.7	6.8	5.5	1.7
	Rank	10	5	6	2	8	3	7	8	4	1
Environment 3	Mean score	5.8	4.7	5.6	5.9	5.9	5.3	5.4	7.8	5.5	3.0
	Rank	7	2	6	8	8	3	4	10	5	1
Environment 4	Mean score	7.7	5.2	4.3	5.0	6.5	5.6	5.0	5.9	6.4	3.4
	Rank	10	5	2	3	9	6	3	7	8	1
Environment 5	Mean score	8.1	4.2	4.8	3.9	8.1	5.2	4.0	6.8	6.1	3.8
	Rank	9	4	5	2	9	6	3	8	7	1
Environment 6	Mean score	6.4	4.7	6.6	5.1	5.4	5.6	6.0	7.6	4.8	2.8
	Rank	8	2	9	4	5	6	7	10	3	1

**Table 7:** Cohen’s d effect size between EAB-BES and other comparative algorithms across six environments.

Environments	EAB-BES VS								
	PSO	DE	TLBO	SMA	SSA	TLSMA	CSSA	BES	mBES
Environment 1	0.21	0.26	1.06	0.10	0.21	0.11	0.52	0.16	0.62
Environment 2	1.40	0.93	1.34	0.42	1.15	0.70	1.19	1.06	0.82
Environment 3	0.97	0.73	0.70	0.80	0.90	0.68	0.67	0.78	0.86
Environment 4	1.46	0.77	0.43	0.61	1.05	0.69	0.28	1.09	0.91
Environment 5	2.22	0.59	0.84	0.29	1.56	0.88	0.11	1.14	1.41
Environment 6	1.71	1.33	1.23	0.84	0.92	1.11	0.81	0.98	1.14

## Data Availability

Data will be made available on request.
